# Long-term feeding of Atlantic salmon with varying levels of dietary EPA + DHA alters the mineral status but does not affect the stress responses after mechanical delousing stress

**DOI:** 10.1017/S0007114522000514

**Published:** 2022-12-28

**Authors:** Chandrasekar Selvam, Antony J. Prabhu Philip, Esmail Lutfi, Trygve Sigholt, Birgitta Norberg, Grete Bæverfjord, Grethe Rosenlund, Bente Ruyter, Nini H. Sissener

**Affiliations:** 1 Institute of Marine Research, Boks 1870 Nordnes, Bergen, 5817, Norway; 2 Central Marine Fisheries Research Institute, Kochi, India; 3 Norwegian Institute of Food, Fisheries and Aquaculture Research, Ås, Norway; 4 BioMar AS, Trondheim, Norway; 5 Institute of Marine Research, Austevoll Research Station, Storebø, Norway; 6 Norwegian Institute of Food, Fisheries and Aquaculture Research, Sunndalsøra, Norway; 7 Skretting ARC, Stavanger, Norway; 8 Department of Animal and Aquacultural Sciences, Norwegian University of Life Sciences, Ås, Norway

**Keywords:** Atlantic salmon, EPA, DHA, Fatty acids, Cortisol, Stress

## Abstract

Atlantic salmon were fed diets containing graded levels of EPA + DHA (1·0, 1·3, 1·6 and 3·5 % in the diet) and one diet with 1·3 % of EPA + DHA with reduced total fat content. Fish were reared in sea cages from about 275 g until harvest size (about 5 kg) and were subjected to delousing procedure (about 2·5 kg), with sampling pre-, 1 h and 24 h post-stress. Delousing stress affected plasma cortisol and hepatic mRNA expression of genes involved in oxidative stress and immune response, but with no dietary effects. Increasing EPA + DHA levels in the diet increased the trace mineral levels in plasma and liver during mechanical delousing stress period and whole body at harvest size. The liver Se, Zn, Fe, Cu, and Mn and plasma Se levels were increased in fish fed a diet high in EPA + DHA (3·5 %) upon delousing stress. Furthermore, increased dietary EPA + DHA caused a significant increase in mRNA expression of hepcidin antimicrobial peptide (HAMP), which is concurrent with downregulated transferrin receptor (TFR) expression levels. High dietary EPA + DHA also significantly increased the whole-body Zn, Se, and Mn levels at harvest size fish. Additionally, the plasma and whole-body Zn status increased, respectively, during stress and at harvest size in fish fed reduced-fat diet with less EPA + DHA. As the dietary upper limits of Zn and Se are legally added to the feeds and play important roles in maintaining fish health, knowledge on how the dietary fatty acid composition and lipid level affect body stores of these minerals is crucial for the aquaculture industry.

Feeds for aquacultured Atlantic salmon have changed from essentially a marine-based diet with a protein/fat ratio of 3:2 in the early 90s to a diet with 70 % plant ingredients and protein/fat ratio of approximately 1:1 today^(1)^. The shift in the ingredient composition of the salmon diet has resulted in a reduced level of long-chain *n*-3 fatty acids (FA), EPA (20:5*n*-3) and DHA (22:6*n*-3), and increased *n*-6 FA content. The beneficial effects of EPA and DHA are well documented^(2–4)^, and the dietary contents of these FA are conditionally essential for Atlantic Salmon^(5–8)^. The pooled dietary requirement of the *n*-3 FA *α*-linolenic acid, EPA and DHA for salmonids has been reported to range from 10 to 25 g/kg feed depending on the species and experimental conditions^(9)^. However, recent results have shown that salmon fed a diet containing 10 g/kg EPA + DHA in the feed throughout the whole production cycle had significantly higher mortality than salmon fed 16 g/kg EPA + DHA when the fish were subjected to repeated handling stress such as delousing at high water temperatures in sea cages^(6)^. In comparison, 11 g of EPA + DHA /kg feed seemed to be sufficient for salmon during long-term feeding during the seawater phase in land-based tanks, and despite some negative health effects, even salmon fed only 5 g of EPA + DHA/kg feed had survival rates ≥ 99 %^(10)^. This clearly shows that the robustness of salmon fed low dietary EPA and DHA needs to be tested under challenging conditions experienced by the fish in sea cages rather than the controlled and stable conditions of land-based tanks. The salmon lice (*Lepeophtheirus salmonis*) are naturally occurring injurious ectoparasites that cause direct injury to sea-farmed salmon and pose a detrimental effect on salmon health and welfare^(11)^. Among the various methods for salmon delousing, thermal and mechanical delousing are the most frequently applied methods for the immediate removal of the salmon lice^(11)^. These delousing procedures involve crowding, handling, transportation or confinement, thereby creating a series of stressful conditions resulting in direct physical/mechanical injury to gills, fins, eyes, skin, etc., which might cause a considerable challenge to fish welfare^(11,12)^.

Besides the change in dietary FA profile, increased inclusion of plant ingredients also reduces the supply and availability of dietary minerals to salmonids^(13)^. Both animal model studies and *in vitro* cell model studies have identified a possible relationship between EPA + DHA supplements and mineral homoeostasis. A significant interaction of EPA + DHA supplements on the expression of selenoproteins^(14)^, Zn transporters^(15)^ and HAMP (hepcidin antimicrobial peptide)^(16)^ were previously reported *in vitro*. Conversely, these relationships are poorly studied in fish. The negative effects of low dietary levels of EPA and DHA included reduced tissue integrity of the intestine^(17)^ and increased plasma cortisol levels (both basal and after a stress challenge)^(18)^. In the latter trial, reductions in liver Fe, Zn and Mn were also observed 3 h after stress, with further reductions 24 h after stress (pers. Comm. NH Sissener, IMR). Recent reports point towards a relation between tissue mineral status and endoplasmic reticulum (ER) stress, lipid and antioxidant metabolism in fish^(19,20)^. Furthermore, environmental stressors cause significant oxidative stress, which in turn affects the antioxidant defence system of animals *in vivo* and subsequently increases oxidative damage^(21,22)^. Additionally, increased oxidative stress leads to increased demand for antioxidant minerals such as Se, Zn, Cu and Mn, thereby reducing the concentration of these minerals in circulation^(23–25)^. Although considerable efforts have been made to understand the effects of dietary FA on oxidative stress^(26–28)^, very few studies have evaluated these effects under stressful conditions^(29,30)^. Therefore, the aim of the present study was to investigate the effect of different levels of dietary EPA and DHA on stress responses, trace mineral concentration in plasma and liver, and expression of antioxidant markers in Atlantic salmon during delousing and also long-term effect of these FA on the whole-body mineral status.

## Materials and methods

### Diets and experimental design

This study was part of a long-term feeding trial with five different diets produced by BioMar AS. The experimental design, diets and fish performance have been described in detail in Lutfi *et al.* ^(31)^ and are also presented in Table 1 (9-mm pellet) for a better understanding of the current study. In brief, four different diets were formulated to contain graded levels of EPA + DHA (10, 13, 16 and 35 g/kg in the diet), and the only differences between the diets were the oil blends used to achieve the desired FA composition. Additionally, the fifth diet used in the current study was formulated to contain 13 g/kg EPA + DHA with a reduced total fat content compared with other four diets. The difference in EPA and DHA levels was achieved with different combinations of rapeseed oil and fish oil. Increasing dietary EPA and DHA levels resulted in decreasing levels of 18:1, 18:2*n*-6 and 18:3*n*-3 (Table 2). The average EPA:DHA ratio in the feeds was about 1·1 from start until the fish were about 500 g and then changed to about 1·6 until the stress trial. The experimental diets are referred to in the text according to their percentage of EPA + DHA in the feed (diet 1·0, diet 1·3, diet 1·6, diet 3·5 and diet 1·3 RF (reduced fat)). The experimental diets were produced from a similar dry feed mixture except for the 1·3 RF diet that had higher inclusion of wheat gluten and less oil added. Three different pellet sizes (4, 6 and 9 mm) and specific formulations of experimental feeds (five batches) were used to meet dietary requirements for the different life stages. The diet formulation of the 9-mm pellet given from approximately 1·0 – 2·5 kg is shown in Table 1 and for the other pellet sizes and feed batches are given in Lutfi *et al.* ^(31)^. The analysed proximate and FA composition of all diets are provided in Table 2 for the 9-mm pellets, while the results from the same analyses for the 4- and 6-mm pellets are given in Lutfi *et al.* ^(31)^. The mineral composition of the 9-mm pellet (given from approximately 1·0–2·5 kg) was analysed, and no differences were found between diets (Table 2).

**Table 1.** Formulation and chemical composition of the experimental diets (9-mm pellet size)

Raw material	Dietary EPA + DHA levels				
	Diet 1	Diet 1·3	Diet 1·3 RF	Diet 1·6	Diet 3·5
Rapeseed oil*	25·89	24·34	19·42	22·84	14·89
Guar meal†	20·00	20·00	17·44	20·00	20·00
Soya SPC	11·35	11·35	8·00	11·35	9·80
Wheat‡	10·81	10·81	13·40	10·81	11·51
Pea protein§	10·74	10·74	10·00	10·74	11·25
Fish oil‖	4·29	5·83	5·83	7·32	15·00
Fishmeal¶	5·00	5·00	5·00	5·00	5·00
Maize gluten**	5·00	5·00	5·00	5·00	5·00
Wheat gluten††	2·18	2·18	11·50	2·18	2·78
Mono-calcium phosphate (MCP) 1·32		1·32	1·32	1·31	1·33
Vitamin and mineral premix‡‡	1·53	1·53	1·53	1·53	1·53
Amino acids§§	1·33	1·33	1·28	1·33	1·36
Lucatin Pink 10 %	0·055	0·055	0·055	0·055	0·055
Water change	0·51	0·52	0·24	0·53	0·52
Chemical composition					
Moisture	6·00	6·00	6·00	6·00	6·00
Gross energy (MJ/kg)	25·30	25·30	24·55	25·30	25·27
Crude protein	36·20	36·20	39·83	36·20	36·18
Crude fat	34·71	34·72	30·10	34·72	34·56
Ash	5·51	5·51	5·21	5·51	5·48

* Denmark.

† India.

‡ Denmark.

§ China.

‖ Peru/Denmark.

¶ Peru/Denmark.

** Ukraine.

†† EU.

‡‡ Sweden.

§§ Germany/Korea/China.

Diet 1/diet 1·3/diet 1·6/ diet 3·5, diet codes are set according to their percentage of EPA + DHA in the feed.

One diet labelled as diet 1·3 RF due to its reduced-fat level.

**Table 2.** Fatty acid composition (% of total fatty acids) and mineral composition (mg/kg) of the experimental diets (9-mm pellet size)

	Dietary EPA + DHA levels				
	Diet 1	Diet 1·3	Diet 1·3 RF	Diet 1·6	Diet 3·5
C 14:0	0·96	1·14	1·45	1·56	3·02
C 16:0	7·55	8·01	8·79	8·96	12·41
C 18:0	2·68	2·92	2·86	3·02	3·45
Σ SFA*	12·32	14·60	15·29	15·95	20·92
C 16:1 *n*-7	1·14	1·34	1·72	1·82	3·52
C 18:1 *n*-7	2·97	2·94	2·95	2·90	2·85
C 18:1 *n*-9	47·84	46·08	44·46	43·71	34·61
C 20:1 *n*-9	1·25	1·24	1·24	1·24	1·22
C 22:1 *n*-11	0·13	0·16	0·20	0·24	0·47
Σ MUFA†	55·00	53·53	52·64	52·17	46·48
C 16:2 *n*-6	0·12	0·14	0·19	0·21	0·41
C 18:2 *n*-6	19·13	18·69	17·99	17·50	13·42
Σ *n*-6‡	19·63	19·01	18·42	17·94	14·25
C 18:3 *n*-3	8·17	7·89	7·46	7·44	5·36
C 20:4 *n*-3	0·07	0·07	0·09	0·11	0·18
C 20:3 *n*-3	1·02	0·26	0·34	0·36	0·73
C 20:5 *n*-3	1·95	2·35	3·00	3·22	6·40
C 22:5 *n*-3	0·29	0·35	0·45	0·48	0·96
C 22:6 *n*-3	1·27	1·52	1·91	2·04	3·95
Σ *n*-3§	12·85	12·51	13·48	13·81	17·66
Σ EPA + DHA	3·22	3·86	4·91	5·26	10·35
Σ PUFA	31·91	32·48	31·90	31·75	31·51
EPA/DHA	1·54	1·55	1·57	1·57	1·62
*n*-3/*n*-6	0·65	0·66	0·73	0·77	1·24
Mineral composition mg/kg					
Ca	5844·5	5927·8	5627·3	5960·4	5400·5
Na	1061·4	1080·4	1022·1	1097·9	1052·2
K	6458·1	6361·4	5509·1	6239·9	5131·6
Mg	1502·2	1507·7	1332·1	1470·4	1285·3
P	7446·1	7624·1	7030·6	7469·7	6984·5
Mn	28·2	27·2	27·8	30·0	26·7
Fe	159·5	164·8	146·9	169·2	150·7
Cu	6·8	6·9	6·4	7·1	6·7
Zn	109·0	107·0	104·6	107·3	103·9
Se	0·8	0·8	0·7	0·8	0·8

* Includes 15:0. 17:0. 22:0. 24:0.

† Includes 16:1 *n*-5. 16:1*n*-9. 17:1*n*-7. 18:1*n*-11. 20:1*n*–7. 22:1 *n*–7. 22:1 *n*-9. 22:1 *n*-11. 24:1 *n*-9.

‡ Includes 18:3 *n*-6.

§ Includes 20:3 *n*-3.

Diet 1/diet 1·3/diet 1·6/ diet 3·5, diet codes are set according to their percentage of EPA + DHA in the feed.

One diet labelled as diet 1·3 RF due to its reduced-fat level.

The feeding trial was carried out at Gildeskål Research station (GIFAS) from October 2017 to January 2019. Atlantic salmon post-smolts ( about 115 g) were acquired from a commercial hatchery (Marine Harvest Glomfjord) and transferred to GIFAS research station. After an initial acclimatisation with a standard commercial diet (BioMar), fish with a mean initial weight of about 275 g were randomly distributed into 15 outdoor sea cages (125 m^3^, 5 × 5 × 5 m) with 190 individuals per cage (in triplicates). Fish were fed the experimental diets to apparent satiation once (autumn and winter periods) or twice (spring and summer) a day. The fish were reared under standard farming conditions. The water temperature, salinity and O_2_ (measured at 3 m depth) were recorded daily, and the average water temperature ranged from 3°C in winter to 16°C in summer. Mortality data were recorded throughout the experiment. The experiment was conducted according to the National Guidelines for Animal Care and Welfare published by the Norwegian Ministry of Education and Research (Norwegian Food Safety Authority (FOTS); approval 16 059).

### Sampling

At the start of the experiment, individual fish length and weight were recorded. Sea lice counts were recorded on weekly basis by randomly selecting one cage per week as per Norwegian regulations and standard procedures at GIFAS. The delousing was performed from 12 to 14 August 2018, when the average fish size reached about 2·5 kg. Before delousing, the fish were starved overnight, and mechanical delousing was performed according to GIFAS standard protocol with manual delousing of each fish. In brief, all fish in a cage were transferred to a small well boat and anaesthetised before lice were removed using wet vacuuming with an adapted mouthpiece. After delousing, fish were dropped directly back in the cage. At each sampling, the net of the pen was raised to gather the fish, before fish are caught by a hand net. After netting, fish were immediately anaesthetised in a tub of water to prevent further stress response before the tub was transported from the net pen to the sampling area. During the delousing period, fish were sampled at three different time points relative to acute stress: before delousing (pre-stress), 1 h after and 24 h after delousing stress. At each sampling point, seven fish per cage were killed using overdose of anesthesia (Tricaine Pharmaq, 0·3 g/l). Weight and length were measured on all fish before blood was taken from the caudal vein with vacutainers coated with EDTA. Blood was centrifuged for 7 min at 4000 g to separate plasma and erythrocytes. Erythrocytes were washed thrice in physiological saline. At each sampling, plasma samples were collected from six individual fish per cage and erythrocytes were collected from three individual fish per cage only at pre-sampling. External welfare indicators were recorded for all sampled fish. For gene expression analysis, individual liver samples from six fish per cage were collected and flash-frozen in liquid N_2_ before they were stored in −80°C until analysis. Final sampling was performed at the end of the experiment on January 2019. Pooled whole-body homogenates (fifteen fish per cage) were collected for mineral analysis for this current study (*n* 3).

### Plasma cortisol

Cortisol was extracted from blood plasma by a method modified from Pankhurst & Carragher^(32)^. Briefly, plasma samples (100 µl) were mixed with 1 ml of ethyl acetate, vortexed for 20 s and centrifuged for 3 min at 1870 rpm and 4°C. The organic phase was collected with a Pasteur pipette, before a second extraction with 1 ml of ethyl acetate. The extracts were evaporated in a Speed Vac centrifuge (Savant 1000) for 30 min and dissolved in 1 ml of buffer (phosphate 0·1 M (pH 7·4), 0·4 M NaCl, 1 mM EDTA) by heating (60°C for 10 min) and stored at −20°C until further analysis. The extracted cortisol was measured by ELISA^(33)^. Cortisol EIA Monoclonal antibody (cat; 400 362), Cortisol AchE (acetylcholinesterase) tracer (cat; 10005272) and 96-well microtitre plates-coated Goat Anti-Mouse IG (cat; 400 008) were purchased from Cayman Chemicals. Standard cortisol was purchased from Sigma Aldrich (Sigma reference standards). According to the manufacturer, the primary antibody shows a 100 % cross-reactivity with cortisol, 0·23 % with 11-deoxycorticosterone and 17-hydroxyprogesterone, 0·15 % with cortisol glucuronide, and 0·14 % corticosterone. The lower detection limit was 0·031 ng/ml. The accepted inter-assay CV was 10 %. The percentage recovery of cortisol was 80 % and final cortisol concentration was corrected according to percentage recovery.

### Gene expression analysis by quantitative real-time PCR

The gene expression analysis was studied in three selected dietary groups, including low (diet 1), mid (diet 1·6) and high (diet 3·5) EPA + DHA levels. Candidate genes involved in trace mineral metabolism (HAMP; TFR, transferrin receptor; Met-B, metallothionein-B), stress response (CAT, catalase; SOD, superoxide dismutase; Gpx1, glutathione peroxidase 1; Gpx4b, glutathione peroxidase 4b; Gpx7, glutathione peroxidase 7; GR, glutathione reductase; GST1, glutathione S-transferase 1; FAS, fatty acid synthase; G6PD, glucose-6-phosphate dehydrogenase; SePP, selenoprotein P and HSP70, heat shock protein 70) and inflammatory markers (IFN-*γ*, interferon gamma; TNF1*α*, tumour necrosis factor 1 alpha; TGF-*β* 1, transforming growth factor *β* 1 and IL4/13a, interleukin 4/13a) were analysed in the liver. The procedure for RNA extraction, reverse transcription and quantitative PCR (qPCR) followed were as described in Hundal *et al.* ^(34)^. In brief, the total RNA was extracted from liver tissue using EZ1 RNA Universal Tissue Kit (Qiagen) and the BioRobot EZ1 according to the manufacturer’s descriptions. Quality and integrity of RNA were assessed with the NanoDrop ND-1000 UV–Vis Spectrophotometer (NanoDrop Technologies) and the Agilent 2100 Bioanalyzer (Agilent Technologies). A two-step real-time PCR protocol was followed to assess the mRNA transcriptional levels of the selected target genes. The stability of the reference genes (geometric mean of both *β*act and elf1*α*) and mean normalised expression of the target genes were calculated using CFX Maestro software (Bio-Rad CFX maestro version 1.1, Bio-Rad laboratories). The details of the qPCR primers used for amplification of the reference and target genes are provided in Table 3.

**Table 3.** Primers used for quantitative PCR

Primers	Forward	Reverse	Accession number
CAT	CCAGATGTGGGCCGCTAACAA	TCTGGCGCTCCTCCTCATTC	NM_001140302·1
SOD	GTTTCTCTCCAGCCTGCTCTAAG	CCGCTCTCCTTGTCGAAGC	XM_014145196·1
Gpx1	TCTCCTGCCATAACGCTTGA	GTGATGAGCCCATGGCCTTA	EH033571
Gpx4b	GGGCAGGTGGTGAAGAGATA	CACGCTAGGTTTCATCAGGC	BT044014·1
Gpx7	TGGGAAAGTCATGGATGCCT	GCTCAGGGTGTTTTGTTGCA	NM_001140889·2
GR	CCAGTGATGGCTTTTTTGAACTT	CCGGCCCCCACTATGAC	XM_014199133·1
GST 1	ATTTTGGGACGGGCTGACA	CCTGGTGCTCTGCTCCAGTT	BT049972·2
SePP	CACCTTCACACCTTGCTGAG	CAGTCCCCACAGATGCTTTG	BT072527·1
Met-B	TGAATAAAGAAGCGCGATCAAA	CTGGTGCATGCGCAGTTG	BT047801·1
HAMP	CATTGAAAATCGTGCATTGG	AAGGCCTTCATTCTCGGTTT	NM_001140849·1
TFR	TTGTCGCAACCCCTATAACC	AAGACAGCCCACATCAGGTC	XM_014188394·1
FAS	CATTGCCATGACGATGTCATAAT	TAAACGCTGACGCCTCATTG	XM_014179800·1
G6PD	GAGCTGCATGATGACAAGGA	TGTTCAGGGAGGAGACCATC	NM_001141724·1
HSP 70	CATCGACTTCTACACCTCCATCAC	CTGAAGAGGTCGGAACACATCTC	BG933934
IFN*γ*	GATGGGCTGGATGACTTTAGGATG	CCTCCGCTCACTGTCCTCAAA	AY795563·1
TNF*α*	GTGTATGTGGGAGCAGTGTT	GAAGCCTGTTCTCTGTGACT	NM_001123617·1
TGF-*β* 1	CTCACATTTTACTGATGTC	GGACAACTGCTCCACC TTGTG	XM_014129261·1
IL4/13a	CCACCACAAAATGCAAGGAGTTCT	CCTGGTTGTCTTGGCTCTTCAC	NM_001204895·1
EF1*α*b	TGCCCCTCCAGGATGTCTAC	CACGGCCCACAGGTACTG	AF321836
*β*-actin	CCAAAGCCAACAGGGAGAA	AGGGACAACACTGCCTGGAT	BG933897

CAT, catalase; SOD, superoxide dismutase; Gpx1, glutathione peroxidase 1; Gpx4b, glutathione peroxidase 4b; Gpx7, glutathione peroxidase 7; GR, glutathione reductase; GST, glutathione S-transferase; SePP, selenoprotein P; Met-B, metallothionein-B; HAMP, hepcidin antimicrobial peptide; TFR, transferrin receptor; FAS, fatty acid synthase; G6PD, glucose 6 phosphate-1 dehydrogenase; HSP 70, heat shock protein 70; IFN-*γ*, interferon gamma; TGF-*β* 1, transforming growth factor beta 1; IL4/13a, interleukin4/13a; EF1*α*b, elongation factor alpha b.

### Mineral analysis

The concentration of minerals in diets, liver, plasma (from delousing stress sampling) and whole fish (from final sampling) were determined by inductively coupled plasma MS (ICP-MS) as described elsewhere^(35)^. Briefly, finely ground samples of the feeds and freeze-dried homogenates of whole fish (approximately 0·5 g) or plasma (0·5 ml) were digested using 10 ml of HNO3 (69 % w/w) and 10 ml of H2O2 (30 % w/w) in an UltraClave (Milestone Inc.). The digested samples were subsequently diluted to 50 ml with Milli-Q® water. The samples were subsequently introduced into the nebuliser tube of the ICP-MS (iCapQ ICP-MS, Thermo Scientific) equipped with an auto sampler (FAST SC-4Q DX, Elemental Scientific), and the elements were detected at corresponding mass-to-charge ratios.

### Fatty acids analysis

FA composition of erythrocytes was analysed using ultra-fast gas chromatography, as described by Sissener *et al.* ^(7)^. In this method, MUFA is not separated according to the position of their double bond and these FA are stated as 16:1, 18;1, 20:1 and 22:1. In brief, samples were thawed and weighed. Nonadecanoic acid (19:0) was added as an internal standard to the samples, and then the samples were saponified and methylated by adding 1 ml NaOH (0·5M) and 2 ml BF_3_ in methanol. The samples were evaporated and then purified with hexane. The final concentration of the samples was adjusted to 0·2–0·3 mg/ml and injected into FA detection system. The system used for FA detection was a Trace GC Ultra (Thermo Corporation) with SSL injector, flame Ionization Detector, and the column was a Wax column (P/N UFMC00001010501, 5-m long, 0·1-mm. Id., 0·1-μm film thickness). Chromeleon^®^ version 7.2 was the integrator used (Thermo Scientific).

### Evaluation of welfare indicators and X-ray analysis of vertebrae

The external welfare indicators (eye cataract, skin lesions, snout damage, and fin damage, including dorsal, pectoral and caudal fins) of fish exposed to delousing stress were evaluated by using a scoring system developed by Nofima and BioMar^(36)^ and also described in details in Lutfi *et al.* ^(31)^. In short, each of the welfare indicators was scored between 0 and 3 where the lowest value represents a good and the highest a poor condition of the fish.

The X-ray radiographic analysis of fish vertebrae was performed at the Nofima X-ray radiography laboratory in Sunndalsøra. The X-ray set-up was semi-digital, with a standard X-ray source (Shimadzu mobile art) and with the exposure (40 kV and 40 mAs) of reusable image plates. The X-ray radiographs were transferred to the computer as digital images and were analysed visually, and variations in bone structures were recorded and classified in a blind evaluation. The number of samples examined was 22–25 per diet. A detailed description of the X-ray analysis was given in Bou *et al.* ^(6)^.

### Data analysis and statistics

Data were tested for homogeneity of variance and normality using a Kolomogorov–Smirnov test and Shapiro–wilk test, respectively. Data from gene expression analysis were log-transformed before statistical analysis. Data from plasma cortisol, plasma and liver trace minerals and gene expression analysis were subjected to a two-way ANOVA, with diet and delousing stress as the two factors. Only in those cases where a significant effect was observed within a factor, nested one-way ANOVA followed by Tukey’s multiple comparisons were performed for each factor separately (*n* = 6/cage). One-way ANOVA followed by Tukey’s multiple comparison were performed for whole-body mineral status (*n* = 3). For all statistical tests, *P*-values < 0·05 were considered significant. All results are expressed as mean ± standard error. Statistica 13.4 (Statsoft Inc.) and GraphPad Prism version 8.0 (Graphpad Software Inc.) were used in the statistical analyses.

## Results

### Performance

The results of growth performance including fish weight, length, specific growth rate and condition factor K were measured from the start of the experiment and until the mid-sampling/delousing sampling and are presented in Table 4. The average body weight increased from about 0·27 to about 2·5 kg during this period with no significant differences in weight, length, specific growth rate and condition factor K between different dietary groups at this time point. As previously stated, the growth performance, FA and other lipid data from the final sampling are reported elsewhere^(31)^. The average size of the fish at the final sampling was about 5 kg. Fish that received a diet containing 3·5 % of EPA + DHA had significantly (*P* < 0·001) higher final weight compared with other dietary groups.

**Table 4.** Growth performance of Atlantic salmon fed experimental diets (until mid-sampling)

	Diet 1		Diet 1·3		Diet 1·3 RF		Diet 1·6		Diet 3·5		ANOVA *P*
	Mean	sem	Mean	sem	Mean	sem	Mean	sem	Mean	sem	
Initial weight (g)	275·4	1·40	275·2	2·88	274·3	1·3	276·4	0·6	277·1	0·7	NS
Mid sampling weight (g)†	2434·9	43·20	2552·8	54·8	2630·6	52·2	2554·9	50·3	2613·8	59·4	NS
Mid sampling length (cm)†	55·67	0·30	56·39	0·5	56·9	0·4	56·6	0·4	57·3	0·4	NS
Final weight (g)¤	4748^a^	33·4	4938^a^	85·5	5100^a^	124·0	4964^a^	68·4	5365^b^	56·9	<0·001
Condition factor K†	1·4	0·01	1·41	0·014	1·42	0·013	1·4	0·01	1·37	0·008	NS
SGR†	0·72	0·01	0·73	0·003	0·75	0·005	0·73	0·004	0·74	0·008	NS
Survival %†,*	100	0·0	99·7	0·0	99·2	0·0	100	0·0	100	0·0	NS

NS, Non-significant; SGR, Specific growth rate.

Data are shown as mean values with their standard errors (*n* = 3).

Statistical significance analysed through one-way ANOVA followed by Tukey’s multiple comparisons.

Significantly different means are denoted by different superscript letters.

Diet 1/diet 1·3/diet 1·6/ diet 3·5, diet codes are set according to their percentage of EPA + DHA in the feed.

One diet labelled as diet 1·3 RF due to its reduced-fat level.

† Data from mid sampling = Delousing sampling.

¤ Final weight of the fish at harvest size.

* Survival percentage presented for the the period of two weeks after delousing.

### The fatty acid composition of the erythrocytes

The FA composition of the erythrocytes as affected by dietary treatments is presented in Table 5. Dietary effects were seen for total *n*-3 FA, which increased with increasing dietary EPA + DHA. Particularly, DHA levels were significantly increased with increasing dietary EPA + DHA, while no difference was seen for EPA. The contrary was seen for MUFA and total *n*-6 FA, and these FA were decreased with increasing dietary EPA + DHA. Particularly, fish fed higher dietary EPA + DHA (diet 3·5) had a significantly lower percentage of MUFA compared with all other dietary groups. The ratio of *n*-3/*n*-6 FA was significantly increased in response to increasing dietary EPA + DHA levels, and high *n*-3/*n*-6 ratio (6·1:1) was seen in the fish fed diet 3·5. The SFA levels in the erythrocytes were not significantly different among different dietary groups.

**Table 5.** Fatty acid composition (percentage of total fatty acids) of erythrocytes of Atlantic salmon fed experimental diets (at pre-delousing stress)

	Diet 1		Diet 1·3		Diet 1·3 RF		Diet 1·6		Diet 3·5	
	Mean	sem	Mean	sem	Mean	sem	Mean	sem	Mean	sem
14:0	0·5	0·1	0·9	0·3	1·3	0·5	0·6	0·1	1·0	0·2
16:0	21·6	1·1	20·0	1·1	19·1	0·6	20·0	0·3	21·1	0·5
18:0	11·0	1·0	10·2	0·8	9·5	0·5	11·0	0·3	11·2	0·3
ΣSFA	33·1	2·0	31·1	1·8	30·0	1·3	31·7	0·5	33·3	0·9
16:1*n*-9	0·3^a^	0·05	0·3^a^	0·04	0·5^a,b^	0·04	0·3^a^	0·04	0·5^b^	0·1
18:1*n*-9	11·2^a^	0·6	11·1^a^	0·6	10·2^a^	0·3	10·0^a^	0·3	7·6^b^	0·2
20:1*n*-9	0·8^a^	0·1	0·6^b^	0·1	0·6^a^	0·1	0·5^a,b^	0·04	0·4^b^	0·1
ΣMUFA	12·3^a^	0·5	12·0^a^	0·5	11·3^a^	0·3	10·8^a^	0·4	8·5^b^	0·3
18:2*n*-6 (LA)	5·3^a^	0·2	4·9^a,b,c^	0·2	4·6^b,c^	0·1	4·2^c^	0·2	2·5^d^	0·03
20:2*n*-6	0·8^a^	0·04	0·9^a^	0·1	0·8^a,b^	0·1	0·6^b^	0·03	0·4^c^	0·04
20:3*n*-6	1·3^a^	0·1	1·2^a^	0·1	1·1^a^	0·1	0·7^b^	0·02	0·4^c^	0·04
20:4*n*-6 (ARA)	4·2^a^	0·1	4·4^a^	0·1	4·5^a,b^	0·1	4·5^a,b^	0·1	5·0^b^	0·1
Σ*n*-6	11·6^a^	0·4	11·3^a^	0·3	11·0^a,b^	0·2	10·1^b^	0·2	8·3^b^	0·2
18:3*n*-3 (ALA)	<0·1		<0·1		<0·1		<0·1		<0·1	
20:4*n*-3	1·1^a^	0·1	1·0^a^	0·1	1·0^a^	0·1	0·9^a^	0·04	0·6^b^	0·1
20:5*n*-3 (EPA)	8·8^a^	0·4	9·4^a^	0·5	9·8^a^	0·3	10·2^a^	0·2	10·0^a^	0·2
22:5*n*-3	3·4^a^	0·2	3·8^a,b^	0·1	4·2^b,c^	0·1	3·9^a,b^	0·1	4·6^c^	0·2
22:6*n*-3 (DHA)	29·3^a^	0·9	31·0^a^	1·0	32·2^ab^	0·7	32·2^a,b^	0·5	34·6^b^	0·8
Σ*n*-3	42·9^a^	1·3	45·5^a,b^	1·5	47·6^b,c^	0·9	47·5^bc^	0·5	49·9^c^	0·8
ΣEPA + DHA	38·0^a^	1·1	40·4^a,b^	1·4	42·0^b,c^	0·9	42·3^b,c^	0·5	44·7^c^	0·9
ΣPUFA	54·6^a^	1·6	56·8^a^	1·6	58·6^a^	1·1	57·5^a^	0·4	58·2^a^	0·9
*n*-3/*n*-6	3·7^a^	0·1	4·0^a,b^	0·1	4·3b^c^	0·1	4·7^c^	0·1	6·1^d^	0·1

LA, linoleic acid; ARA, arachidonic acid; ALA, *α*-linoleic acid; FA, fatty acid.

Data are shown as mean values with their standard errors (*n* = 3).

Different superscript (small letters) indicates statistical significance as obtained through one-way ANOVA followed by Tukey’s multiple comparisons.

Significantly different means are denoted by different superscript letters.

Diet 1/diet 1·3/diet 1·6/ diet 3·5, diet codes are set according to their percentage of EPA + DHA in the feed.

One diet labelled as diet 1·3 RF due to its reduced-fat level.

### Welfare indicators

The mean sea lice count was 0·13 ± 0·027 (mean ± sem) gravid female lice / salmon recorded from the start of the experiment until delousing procedure. The external welfare scores (eye cataract, skin lesions, snout damage, and damages in fins, including dorsal, caudal pectoral, and pelvic fins) were recorded during the delousing stress sampling (Table 6). Neither delousing stress nor diet caused any significant difference among external welfare scores. X-ray radiography analysis of vertebra showed few fish having specific pathological lesions in the spine, that is, vertebral fusions were recorded for 14 %, and cross-stitch vertebra was recorded for 7 % of total analysed fish. Despite some differences in the observed values, dietary effects were not statistically significant, neither in the percentage of affected fish nor in the extent of lesions, including fusion vertebra and across-stitch vertebra (Table 7).

**Table 6.** Visual evaluation of external welfare indicators during delousing period (irrespective of stress conditions)

	Diet 1		Diet 1·3		Diet 1·3 RF		Diet 1·6		Diet 3·5		ANOVA *P*
	Mean	sem	Mean	sem	Mean	sem	Mean	sem	Mean	sem	
Skin	0·9	0·1	1·1	0·1	1·0	0·0	1·1	0·1	1·0	0·0	NS
Eye damage	0·1	0·0	0·1	0·0	0·1	0·0	0·1	0·1	0·1	0·0	NS
Snout	0·1	0·5	0·2	0·4	0·3	0·5	0·3	0·6	0·4	0·5	NS
Dorsal fin	1·1	0·1	3·1	2·1	0·9	0·1	1·1	0·1	1·0	0·0	NS
Caudal fin	1·0	0·0	1·0	0·0	1·0	0·0	1·1	0·0	1·0	0·0	NS
Pectoral fin	1·0	0·0	0·9	0·1	1·0	0·0	0·9	0·0	0·8	0·1	NS
Pelvic fin	0·5	0·1	0·6	0·1	0·5	0·1	0·6	0·1	0·5	0·1	NS

Data are shown as mean values with their standard errors (*n* = 3).

Statistical significance analysed through one-way ANOVA followed by Tukey’s multiple comparisons.

Significantly different means are denoted by different superscript letters.

Diet 1/diet 1·3/diet 1·6/ diet 3·5, diet codes are set according to their percentage of EPA + DHA in the feed.

One diet labelled as diet 1·3 RF due to its reduced-fat level.

**Table 7.** X-ray radiography analysis of vertebra showed from pre-delousing time point

	Diet 1	Diet 1·3	Diet 1·3 RF	Diet 1·6	Diet 3·5	Total sum	ANOVA *P*
Number of fish analysed	23	26	23	21	21	114	NS
Fish with fusion (*n*)	3	5	2	5	1	16	NS
Fish with fusion	13	19	9	24	5	14	NS
Fish with cross-stitch lesions (*n*)	1	3	1	1	1	7	NS
Fish with cross-stitch lesions	4	12	4	5	5	6	NS

Data are shown as mean values with their standard errors (*n* = 3).

Statistical significance analysed through one-way ANOVA followed by Tukey’s multiple comparisons.

Significantly different means are denoted by different superscript letters.

Diet 1/diet 1·3/diet 1·6/ diet 3·5, diet codes are set according to their percentage of EPA + DHA in the feed.

One diet labelled as diet 1·3 RF due to its reduced-fat level.

### Plasma cortisol

**Fig. 1.**
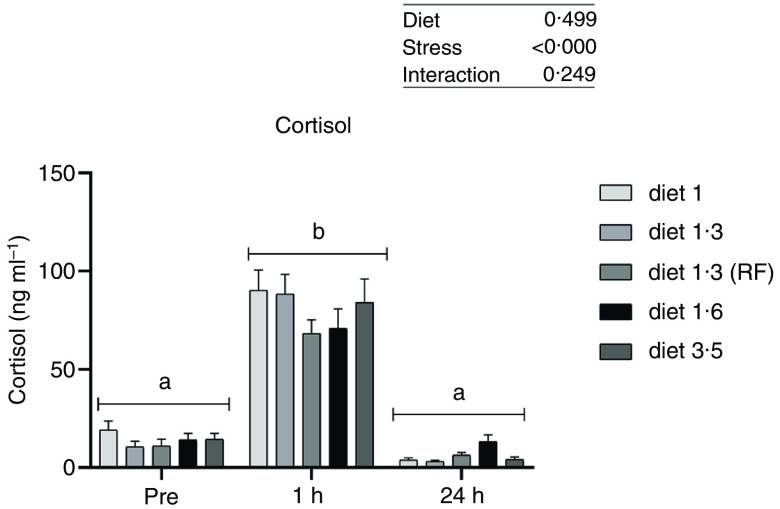
Plasma cortisol (ng/ml) in Atlantic salmon subjected to delousing stress. Fish were sampled before delousing stress (0 h) and 1 h and 24 h post-delousing stress. Small letters (a, b and c) indicate the statistical difference in cortisol levels between time points detected with two-way ANOVA followed by Tukey’s multiple comparisons. Nested one-way ANOVA was performed and no significant difference between dietary groups were detected at any of the sampling points. All data are shown as mean ± sem, *n* = 3. RF, reduced fat.

Plasma cortisol, a primary stress response marker, was measured before, 1 h after and 24 h after delousing stress (Fig. 1). The cortisol responses were not significantly affected by diet at any sampling point, and no interaction effect between diet and stress on cortisol levels was found. As expected, plasma cortisol levels were dramatically increased 1 h post-delousing stress (*P* < 0·0001) and then decreased and returned to pre-stress levels within 24 h, with no statistical differences observed between pre- and 24 h post-stress.

### Plasma trace mineral levels

**Fig. 2.**
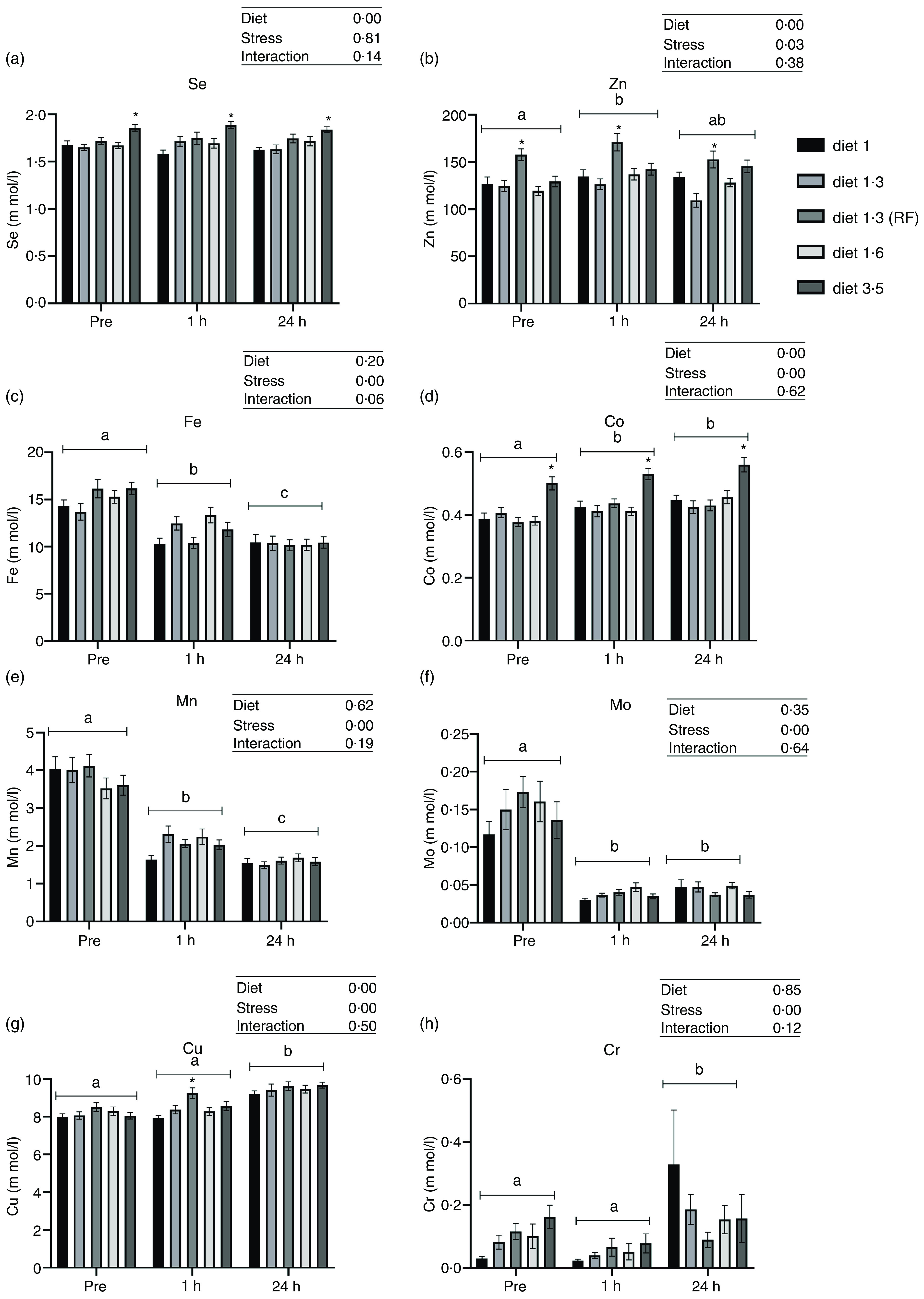
Plasma trace minerals in Atlantic salmon subjected to delousing stress. Fish were sampled before delousing stress (pre-stress) and 1 h and 24 h post-stress. Small letters (a, b and c) indicate the statistical difference between stress conditions detected with two-way ANOVA followed by Tukey’s multiple comparisons. Asterisks (*) indicate the statistical difference between dietary groups detected in nested one-way ANOVA followed by Tukey’s multiple comparisons. All data are shown as mean ± sem, *n* = 3. RF, reduced fat.

Dietary EPA + DHA levels had a significant impact on plasma Co, Cu, Zn and Se levels (Fig. 2). Significant increase in plasma Zn level was observed in fish fed with diet 1·3 RF compared with the other dietary groups (diet 1·0, diet 1·3, diet 1·6 and diet 3·5) (*P* < 0·00001), irrespective of stress factors. Similarly, Cu level was also increased in fish fed with diet 1·3 RF compared with other dietary groups (*P* = 0·0006); however, this difference was observed only 1 h post-stress. Fish fed with high dietary EPA + DHA level (diet 3·5) had significantly increased Co level compared with other dietary groups, irrespective of stress factors (*P* = 0·0001). As there was no stress effect for Se, all the values were nested under each diet group irrespective of stress condition for one-way analysis. Significant dietary effects were found for plasma Se levels. Fish fed high EPA + DHA (diet 3·5) had significantly increased plasma Se level compared with diet 1·0 and diet 1·3 (*P* < 0·0001). No significant difference in plasma Se was found between low-fat (diet 1·3 RF) and high-fat fed group (diet 1·3). A significant change in most analysed trace mineral levels (Cr, Mn, Fe, Co, Cu and Zn) were observed after delousing stress, except for Se (Fig. 2). Mn and Fe levels were significantly decreased at 1 h post-stress compared with pre-stress, and these levels were further decreased at 24 h post-stress (Mn, *P* < 0·0001; Fe, *P* < 0·00001). On the other hand, Co and Zn levels were increased after stress compared with pre-stress conditions. Zn and Co levels were increased at 1 h post-stress, and 24 h post-stress, Co levels remained elevated, whereas Zn level decreased back to pre-stress level (Co, *P* < 0·0001; Zn, *P* = 0·03). Similarly, plasma Cu levels were also increased at 24 h post-stress compared with pre- and 1 h post-stress conditions (*P* = 0·0001).

### Liver trace mineral levels

**Fig. 3.**
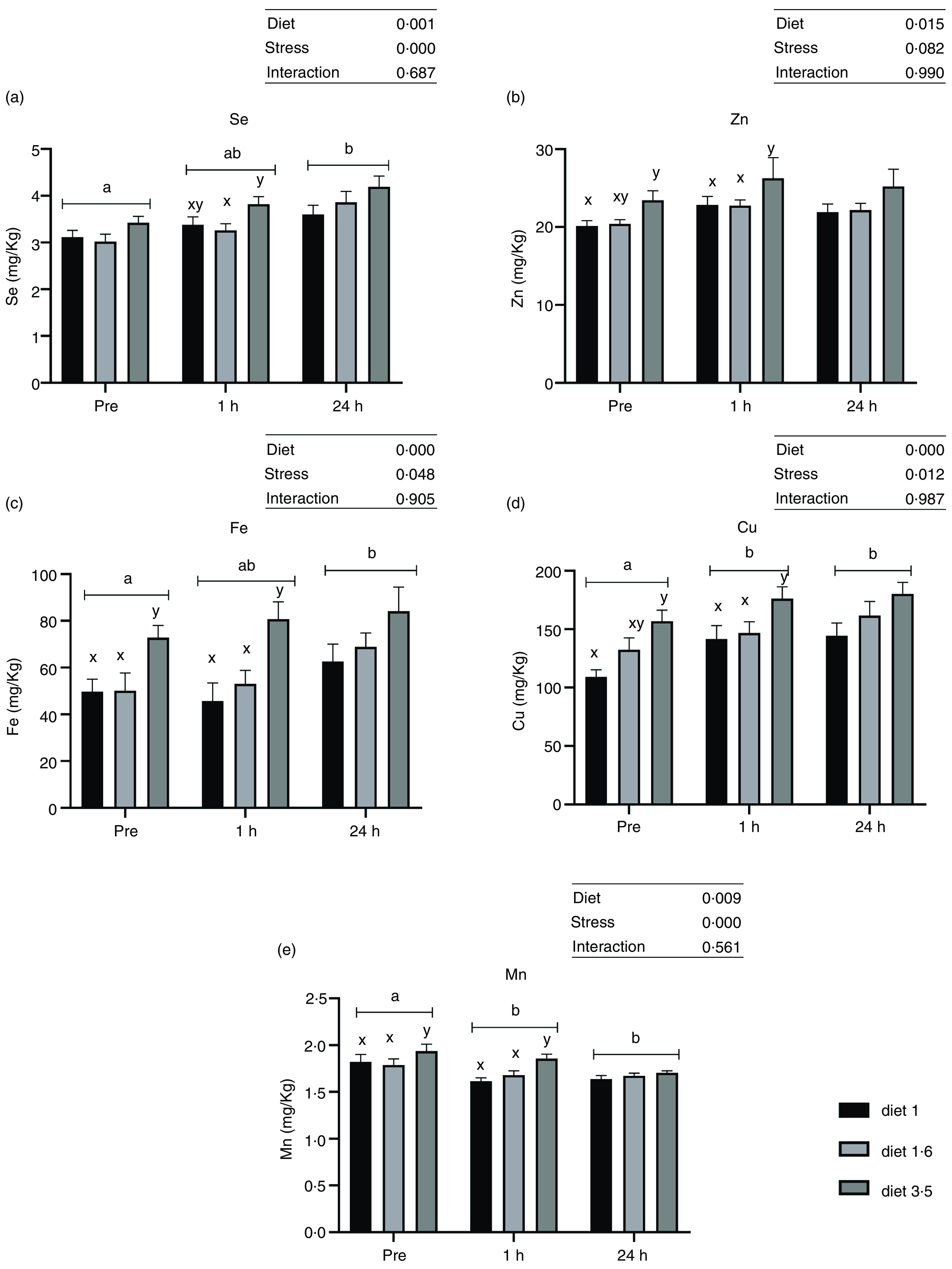
Liver trace minerals in Atlantic salmon subjected to delousing stress. Fish were sampled before delousing stress (pre-stress) and 1 h and 24 h post-stress. Small letters (a, b and c) indicate the statistical difference between stress conditions detected with two-way ANOVA followed by Tukey’s multiple comparisons. All data are shown as mean ± sem, *n* = 3.

Liver trace mineral (Se, Zn, Fe, Cu and Mn) levels during delousing stress were analysed in three selected dietary groups, which include low (diet 1·0), mid (diet 1·6) and high (diet 3·5) EPA + DHA. There was a significant effect of diet on liver trace minerals (Se, *P* = 0·001; Zn, *P* = 0·015; Fe, *P* < 0·0001; Cu, *P* < 0·0001; and Mn, *P* = 0·009; Fig. 3). Irrespective of stress conditions, liver Se, Zn, Fe, Cu and Mn were significantly increased in fish that received high EPA + DHA diet (diet 3·5) compared with the two other dietary groups, while no significant difference was observed between diet 1·0 and diet 1·6. Similarly, liver trace mineral levels were significantly affected by delousing stress. Se, Fe and Cu levels were increased at 24 h post-stress compared with pre-stress, and for Cu this difference was observed already 1 h post-stress. However, Mn level was decreased at 1 h and 24 h post-stress compared with pre-stress. The Zn level remained the same in all stress conditions. There were no ‘diet*stress’ interactions observed for any mineral levels in the liver.

### Liver mRNA expression analysis

**Fig. 4.**
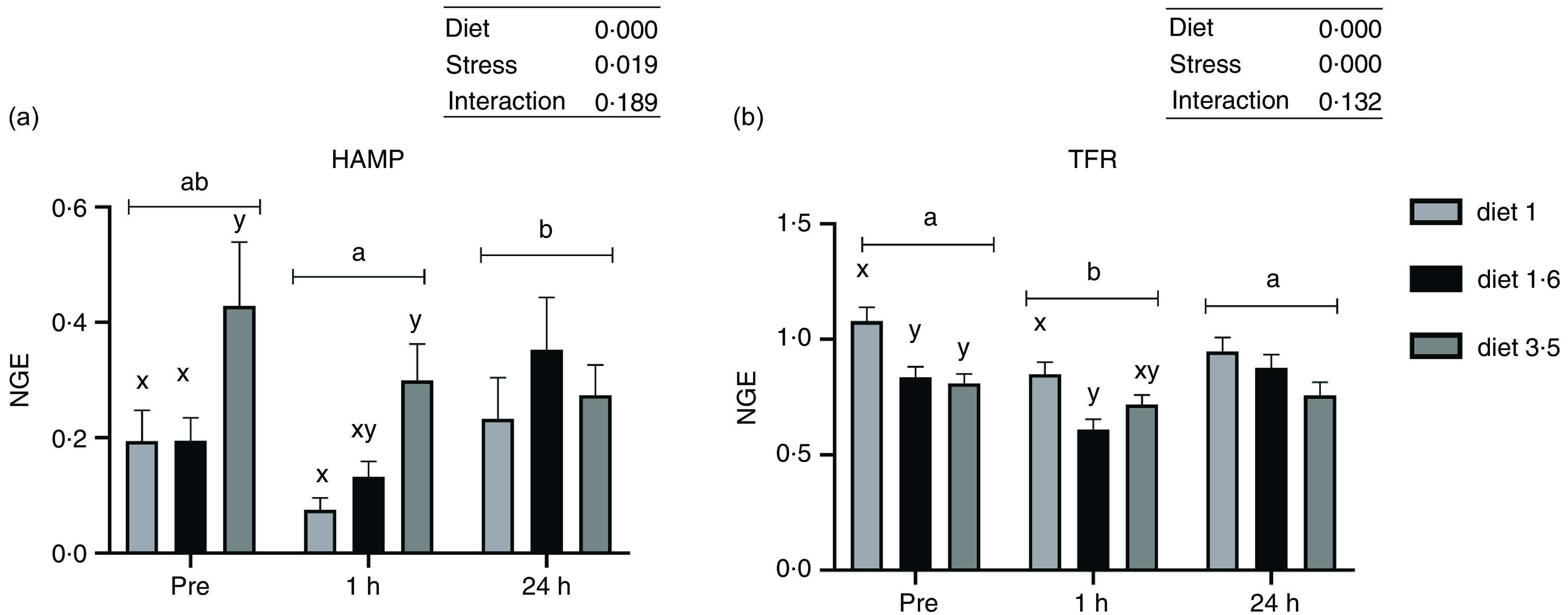
The liver mRNA expression of hepcidin antimicrobial peptide (HAMP, a); transferrin receptor (TFR, b). Fish were sampled before delousing stress (pre-stress) and 1 h and 24 h post-stress. Small letters (a, b and c) indicate the statistical difference between stress conditions detected with two-way ANOVA followed by Tukey’s multiple comparisons. Small letters x, y and z indicate the significant difference between dietary groups detected in nested one-way ANOVA. All data are shown as mean ± sem, *n* = 3. Log-transformed values were used for statistical purposes. NGE, normalised gene expression.

**Fig. 5.**
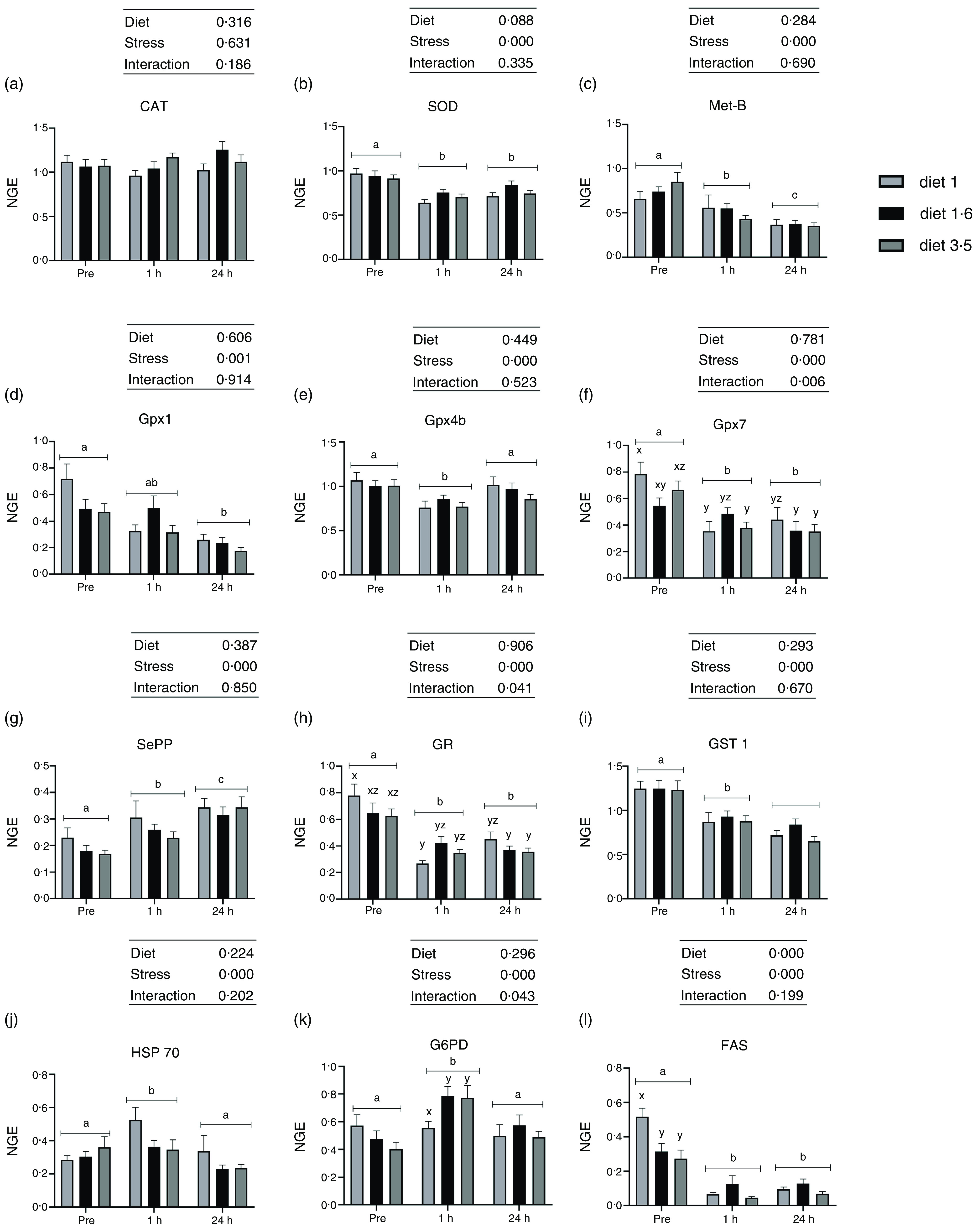
The liver mRNA expression of oxidative stress marker genes in Atlantic salmon subjected to delousing stresses. Catalase (CAT, a); superoxide dismutase (SOD, b); metallothionein B (Met-B, c); glutathione peroxidase (Gpx1, d; Gpx4b, e; Gpx7, f); selenoprotein P (SePP, g); glucocorticoid receptor (GR, h); glutathione S-transferase (GST, i); heat shock protein 70 (HSP 70, j); glucose-6-phosphate 1-dehydrogenase (G6PD, k); fatty acid synthase (FAS, l). Fish were sampled before delousing stress (pre-stress) and 1 h and 24 h post-stress. Small letters (a, b and c) indicate the statistical difference between stress conditions detected with two-way ANOVA followed by Tukey’s multiple comparisons. Small letters x, y and z indicate the statistical difference between dietary groups detected in nested one-way ANOVA. All data are shown as mean ± sem, *n* = 3. Log-transformed values were used for statistical purposes. NGE, normalised gene expression.

The mRNA expression of genes involved in stress response and trace mineral metabolism was analysed (Fig. 4 and 5). The mRNA expression of the HAMP and TFR was significantly influenced either in response to dietary EPA + DHA or delousing stress (Fig. 4). Fish fed high EPA + DHA diet (diet 3·5) had significantly increased HAMP mRNA expression compared with other dietary groups (diet 1·0 and diet 1·6) at pre-stress (*P* = 0·03) and 1 h post-stress (*P* = 0·02). There was no statistical difference observed for diet at 24 h post-stress (*P* = 0·25). Additionally, there was a significant stress effect; HAMP mRNA expression was significantly downregulated at 1 h post-stress compared with pre-stress and 24 h post-stress, and no significant difference was detected between pre-stress and 24 h post-stress. The mRNA expression of TFR was significantly downregulated 1 h post-stress compared with pre- and 24 h post-stress, while at 24 h post-stress TFR expression was back to pre-stress level. Significant diet effects were observed for TFR; fish fed low EPA + DHA (diet 1) had increased TFR expression compared with fish fed diet 1·6 and diet 3·5 pre-stress (*P* = 0·025) and only to diet 1·6 at 1 h post-stress (*P* = 0·03), with a similar (non-significant) trend at 24 h post-stress.

No effects of dietary EPA + DHA on mRNA expression of oxidative stress markers (CAT, SOD, Met-B, Gpx1, Gpx4b, Gpx7, SePP, GR, GST 1 and HSP70) were detected at any sampling point (Fig. 5). However, expression of all the analysed oxidative stress marker genes, except CAT, were significantly affected by delousing stress (Fig. 5). The SOD expression was significantly downregulated at 1 h post-stress compared with pre-stress and remained downregulated 24 h post-stress (*P* = 0·0001). Similarly, the mRNA expression of Gpx1, Gpx4b and Gpx7 were also significantly downregulated at 1 h post-stress. However, at 24 h post-stress, Gpx4b was back to pre-stress level, whereas Gpx1 and Gpx7 levels remained downregulated (Gpx1, *P* = 0·001; Gpx4b, *P* = 0·0001 and Gpx7, *P* = 0·0001). The mRNA expression of SePP was upregulated 1 h post-stress and was even further upregulated 24 h post-stress (*P* = 0·0001). The mRNA expression of Met-B was significantly downregulated 1 h post-stress compared with pre-stress and further downregulated 24 h post-stress (*P* = 0·0001). The expression of GR and GST1 were also significantly downregulated 1 h post-stress. However, GR expression remained the same, whereas GST1 was further downregulated 24 h post-stress (*P* = 0·000, GR; *P* = 0·0001, GST1). The mRNA expression of HSP70 was upregulated 1 h post-stress and then back to pre-stress levels 24 h post-stress (*P* = 0·000). Significant interaction between diet and stress was observed for Gpx7 and GR (Gpx7, *P* < 0·006; GR, *P* < 0·04). The mRNA expression of FAS was significantly downregulated at 1 h and 24 h post-stress compared with pre-stress (Fig. 5). A dietary effect was observed pre-stress, where fish fed diet 1·0 had significantly increased FAS mRNA expression compared with the other dietary groups (diet 1·6 and diet 3·5). No dietary effects were seen in FAS mRNA expression 1 h and 24 h post-stress, and no ‘diet*stress’ interaction was detected. The mRNA expression of G6PD was upregulated 1 h post-stress and was back to pre-stress levels 24 h post-stress (*P* = 0·0001). No effects of dietary EPA + DHA levels on mRNA expression of G6PD were detected at any sampling point; however, significant ‘diet*stress’ was observed for G6PD mRNA expression (*P* = 0·043). Fish that received low EPA + DHA had significantly low G6PD mRNA expression compared with other dietary groups at 1 h post-stress, and this difference was not observed in other sampling points.

**Fig. 6.**
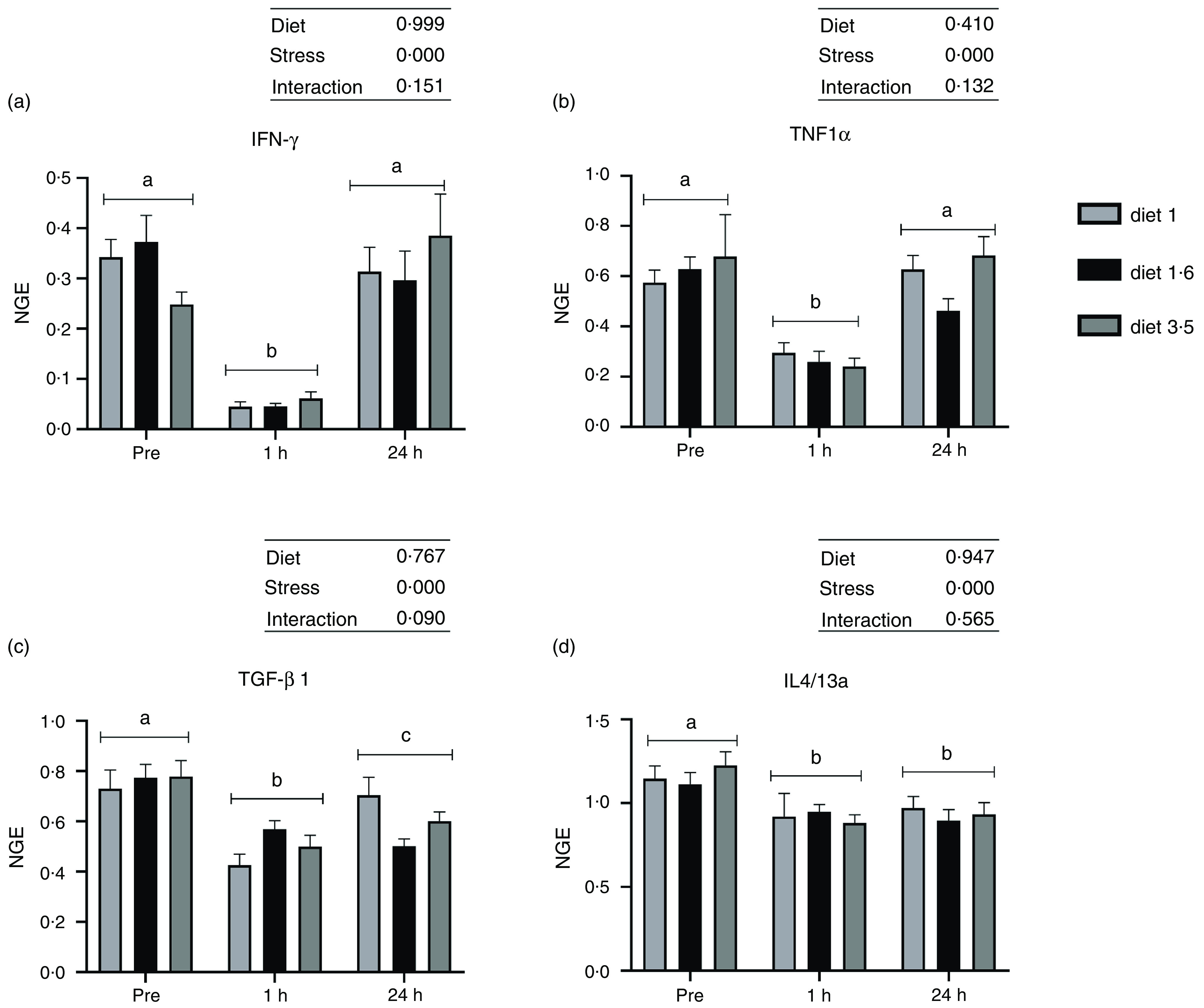
The liver mRNA expression of selected immune genes in Atlantic salmon subjected to delousing stresses. Interferon-gamma (IFN-*γ*, a); TNF1*α*, b; transforming growth factor beta 1 (TGF-*β* 1, c); IL4/13a, d). Fish were sampled before delousing stress (pre-stress) and 1 h and 24 h post-stress. Small letters (a, b and c) indicate the statistical difference between stress conditions detected with two-way ANOVA followed by Tukey’s multiple comparisons. Small letters x, y and z indicate the statistical difference between dietary groups detected in nested one-way ANOVA. All data are shown as mean ± sem, *n* = 3. Log-transformed values were used for statistical purposes. NGE, normalised gene expression.

The mRNA expression of the analysed inflammatory genes (IFN-*γ*, TNF1*α*, TGF-*β* 1 and IL4/13a; Fig. 6) was also significantly downregulated at 1 h post-stress (IFN-*γ, P* < 0·00001; TNF1*α*, *P* < 0·00001; TGF-*β* 1, *P* < 0·00001; IL4/13a, *P* < 0·00002). IFN-*γ* and TNF1*α* expression were back to pre-stress levels 24 h post-stress. However, TGF-*β* 1 and IL4/13a remained downregulated even 24 h post-stress. No significant dietary effects or interaction between diet and stress were observed for these genes.

### Long-term effect – whole-body mineral status

Whole-body trace mineral (Zn, Cu, Mn, Se and Fe) levels were analysed for the fish at the final sampling when the fish reached harvest size (about 5 kg) (Table 8). Higher inclusion of dietary EPA + DHA levels had a significant impact on increasing the whole-body Zn, Mn and Se levels (Zn, *P* = 0·0001; Mn, *P* = 0·01; Se, *P* < 0·0001). The whole-body Zn level was significantly higher for fish that had received high dietary EPA + DHA (diet 3·5) compared with the ones fed diet 1·0, diet 1·3 and diet 1·6, while there was no difference between diet 3·5 and diet 1·3 RF. Further, in fish fed diet 1·3 RF significantly higher body Zn levels were found compared with diet 1·3. The whole-body Se and Mn levels were also significantly higher for fish fed the diet containing 3·5 % of EPA and DHA compared with the ones fed the other diets. The level of Fe and Cu were not significantly different among dietary treatments.

**Table 8.** Analysed whole-body trace mineral concentrations of Atlantic salmon from long-term seawater trial (mg/kg ww)

	Diet 1		Diet 1·3		Diet 1·3 RF		Diet 1·6		Diet 3·5	
	Mean	sem	Mean	sem	Mean	sem	Mean	sem	Mean	sem
Zn	22·7^a^	1·2	23·0^a^	1·0	31·7^b^	3·2	24·0^a^	1·2	33·0^b^	1·5
Cu	1·6^a^	0·0	2·0^a^	0·2	1·6^a^	0·1	1·7^a^	0·1	1·7^a^	0·1
Mn	1·3^a^	0·1	1·4^a^	0·1	1·4^a^	0·0	1·4^a^	0·2	2·3^b^	0·3
Se	0·26^a^	0·001	0·28^a^	0·005	0·25^a^	0·001	0·28^a^	0·003	0·34^b^	0·003
Fe	9·7^a^	0·1	10·2^a^	0·9	9·8^a^	0·1	10·3^a^	0·7	10·0^a^	0·6

Data are shown as mean values with their standard errors (*n* = 3).

Different superscript (small letters) indicates statistical significance as obtained through one-way ANOVA followed by Tukey’s multiple comparisons.

Significantly different means are denoted by different superscript letters.

Diet 1/diet 1·3/diet 1·6/ diet 3·5.

Diet codes are set according to their percentage of EPA + DHA in the feed. One diet labelled as diet 1 3 RF due to its reduced-fat level.

## Discussion

The goal of the present study was to investigate the effects of different levels of EPA + DHA (1·0, 1·3, 1·3 (RF), 1·6 and 3·5 % of the diet) on the stress response in Atlantic salmon undergoing a delousing procedure. As mentioned earlier, the growth performance of salmon fed four of the experimental diets (diet 1, diet 1·3, diet 1·6 and diet 3·5) from the larger feeding trial has been reported elsewhere^(31)^. The minimum requirement for EPA + DHA in Atlantic salmon has been reported to range from 5 to 10 g/kg feed. However, the challenging environmental conditions and other stressors in sea cages, besides other biological factors such as the growth rate of the species, strain disease resistance, etc., are likely to influence this requirement. For example, in a recent long-term seawater trial in Atlantic salmon, Bou *et al.* ^(6)^ reported a significant increase in mortality after delousing at high water temperature in fish fed low 10 g/kg EPA + DHA compared with fish fed 17 g/kg DHA + EPA diet. Another study in Atlantic salmon reared in sea cages under commercial conditions and fed two different levels of EPA + DHA (16 and 26 g/kg feed) showed no difference in mortalities after sea lice treatments and also did not affect the fish performance and robustness^(37)^. However, in the later study, the lowest EPA + DHA (16 g/kg) was close to current commercial level. Nevertheless, in the current study, despite low dietary EPA + DHA in diet 1 (10 g/kg), we did not find any significant mortality after delousing stress. The probable reason for the lack of mortality might be the lower lice infection rate (0·13 gravid female lice / salmon), which was lower than allowed limits (0·5 gravid female lice / salmon) in Norway or the efficient delousing procedure followed during delousing compared with the previously mentioned experiment. In addition, in the current study, mean temperature during delousing was 13°C. In contrast, the delousing in Bou *et al.* ^(6)^ was carried out during a high water temperature period (17·5°C), which might have caused additional stress for the fish. The other possible reason for the lack of mortality could be the previous nutritional history of the animal, as fish in the current trial received commercial diets (17 g/kg EPA + DHA) before the start of the experiment (about 270 g), which was assumed to be sufficient. In addition, neither delousing stress nor diet caused any significant difference in external welfare scores of the fish. Further, although a prominent specific pathological lesion in the spine, that is, fusion vertebrae was recorded in 14 % of analysed vertebrae, but no significant relation to dietary EPA + DHA was found. The type of specific pathological lesion (fused vertebrae) and percentage (14 %) and fused vertebrae observed in this study resembles very much to Bou *et al.* ^(6)^, who reported 18 % fused vertebrae with no effect of low dietary EPA + DHA on vertebral deformities in Atlantic salmon in a long-term trial in sea cages. Furthermore, the fused vertebrae are typical observations in Atlantic salmon produced commercially and it might be due to early smoltification and seawater transfer^(38)^ and may not be coupled with dietary contents. Taken together, data from mortality, external welfare scores and X-ray radiographic analysis of vertebrae indicate that low dietary levels of EPA + DHA (10 g/kg) do not negatively influence the robustness of the fish upon delousing stress.

The increase of plasma cortisol is recognised as a stress indicator^(39,40)^ and was observed 1 h after the delousing procedure, returning to basal level 24 h post-stress. However, the response in cortisol was independent of the dietary EPA + DHA levels. An increased plasma cortisol in response to low *n*-3 LC-PUFA was observed in marine fish in presence or absence of stress^(41–43)^. Furthermore, in fish, it has been shown that eicosanoids derived from LC-PUFA such as EPA and arachidonic acid can modulate the ACTH release from the hypothalamic-pituitary-adrenal (HPI) axis and thereby influence the cortisol production from inter-renal tissue^(44,45)^. However, in Atlantic salmon, different dietary *n*-6/*n*-3 ratio of LC-PUFA (from 1:1 to 6:1) and their absolute contents did not affect plasma cortisol level during acute stress^(34)^. Our results indicate that even the lowest level of EPA + DHA used in the current study was sufficient to mount a cortisol response to acute stress, indicating that this is a highly prioritised physiological function in the fish. The difference to studies in marine fish could be explained by the fact that Atlantic salmon can desaturate and elongate *n*-3 linoleic FA to EPA and DHA^(46,47)^, unlike other marine fish, and further selectively retain them in the membrane when fed with low *n*-3 LC-PUFA^(48,49)^.

FAS catalyses the *de novo* FA synthesis in the presence of NADPH^(50)^. The G6PD is a key regulatory enzyme in the pentose phosphate pathway involved in NADPH production, essential for FA biosynthesis and the maintenance of the redox state in fish and other vertebrates^(51)^. Thus, the increased mRNA expression of G6PD and subsequent high FAS expression during pre-stress in salmon fed low dietary EPA + DHA (diet 1) may indicate the possible up-regulation of FA biosynthesis in response to low *n*-3 LCPUFA diet, as previously described in Atlantic salmon^(52,53)^. Further, after delousing stress, mRNA expression of FAS was significantly downregulated, on the other hand G6PD expression increased dramatically compared with pre-stress levels. This result suggests the possible inhibition of FA biosynthesis under stress conditions or might also indirectly indicate the activation of lipolysis upon energy demand. Further, stress caused increased expression of G6PD, demonstrating the increased NADPH production to maintain the redox status and also to meet the enhanced energy demand during the stressful environment^(54–56)^.

The enzymes SOD, CAT, Gpx and GST play a crucial role in glutathione metabolism and antioxidant defence system by scavenging the reactive oxygen species. The mRNA expression of these genes are recognised as important indicators of oxidative stress^(57)^. Further, metallothionine and selenoproteins are also linked to the glutathione metabolism in the redox cycle^(58)^. Accordingly, the down-regulation of genes in dismutation of the superoxide radical (SOD), glutathione metabolism (Gpx1, Gpx7, GR and GST1) and Met-B 1 h and 24 h post-stress with concurrent up-regulation of SePP was indicative of oxidative stress during delousing^(59–61)^. In Atlantic salmon fed graded levels of *n*-3 LC-PUFA (2·6 to 4·2 % of feed), mRNA expression of antioxidant genes in the liver was not affected^(62)^. In our study, even though the delousing procedure significantly affected the mRNA expression of oxidative stress markers, dietary EPA + DHA levels did not alter their expression either in the presence or absence of stress. However, an increase in HAMP expression, with concurrent down-regulation of TFR and high Fe status in the liver of fish fed the high EPA + DHA diet signifies a pro-oxidant environment. HAMP transcript levels increase with increased Fe load to degrade the Fe exporter ferroportin, which in turn diminish the Fe uptake by acting reciprocally with Fe import proteins such as DMT or TFR^(63)^. Similar increased expression of HAMP in response to DHA supplement was reported in mammals^(64)^. Thus, our results suggest the possible effect of EPA + DHA on the Fe store in fish as reported in mammals^(64)^. However, despite accumulation of a pro-oxidant (Fe) in the liver during delousing, a high dietary EPA + DHA (diet 3·5) does not induce oxidative stress, reiterating that Atlantic salmon is robust to a pro-oxidative environment^(65)^.

Zn and Se are the most limiting trace elements in present-day plant-based salmonid feeds^(13,66)^. Increased use of plant-based ingredients containing anti-nutritional factors like phytic acid reduce Zn availability, thereby reducing their body status. Nevertheless, decreasing dietary EPA + DHA or increasing the fat level can also have consequence on body Zn retention, as shown in mammals. Zn retention in mammals is reduced by high-fat or low EPA + DHA diets thereby increasing the need for oral Zn supplements^(67–69)^. The higher Zn in the whole body of salmon fed either high EPA + DHA (diet 3·5) or low dietary fat (diet 1·3 RF) indicate an interaction between dietary fat or LC-PUFA and Zn in the diet. In this regard, the increase of dietary fat up to 38 % in salmon feeds over the years, and simultaneous decrease in body Zn status in ready-to-slaughter salmon (4–5 kg size fish; 55 to 35 mg/kg)^(1,70)^, warrant further attention. Similarly, increased inclusion of EPA + DHA in the diets markedly increased the plasma and whole-body Se levels in salmon fed diet 3·5. Even though the dietary Se levels satisfied the known minimum requirements of salmon, stressors can affect Se utilisation^(24,25,35)^. In the present study, the increased Se status with 3·5 diet might indicate improved stress mitigation and could in turn protect *n*-3 LC-PUFA from oxidation.

The beneficial effects of dietary *n*-3 LC-PUFA on immune functions are well documented in many vertebrates^(2–4)^, including Atlantic salmon^(5,6,8)^. However, many studies in Atlantic salmon have found no detrimental effects on health and immune functions when fed low dietary *n*-3 LC-PUFA^(71–73)^. Similarly, in this current study, dietary EPA + DHA level did not alter the mRNA expression of analysed immune genes (IFN-*γ*, TNF-*α*, TGF-*β* and IL4/13a). As pointed out by others^(71,73,74)^, the dietary manipulation of gene expression is not always straightforward. It may also be influenced by various other factors, which include ingredient composition, duration of feeding, species, studied tissue, rearing conditions and other environmental factors. Further, the delousing stress significantly downregulated the mRNA expression of all analysed immune genes (IFN-*γ*, TNF-*α*, TGF-*β* and IL4/13a) at 1 h post-stress, which is concurrent with increased cortisol production. The similar down-regulation of immune response upon stress exposure has been reported previously in mammals^(75,76)^ and in fish^(77,78)^.

In conclusion, although delousing causes considerable stress, dietary EPA + DHA levels and fat levels did not affect stress responses in Atlantic salmon. Further, it was noteworthy that increasing EPA + DHA levels in the diet increased the Se and Zn status in plasma and the whole body, while dietary fat level affected Zn status. As these minerals are crucial to fish health, and upper limits exist for their addition in feeds, knowledge on how other dietary factors affect their uptake and retention are of high relevance to the aquaculture industry. However, further studies are necessary in order to elucidate the underlying mechanism for how dietary FA and dietary lipid level affects body mineral status in fish.
